# Supplementary material for: Development of a computer-aided design software for the quantitative evaluation of aesthetic damage
Source: PLoS One. 2019 Dec 18;14(12):e0226322. doi: 10.1371/journal.pone.0226322 (PMC6919621; doi:10.1371/journal.pone.0226322)
Supplement: S1 Code site — 1. (ZIP) [file pone.0226322.s001.zip › [EESC_jr][Projeto]Relatorio_07_21/reports/templates_zip/gentelella-master/vendors/google-code-prettify/styles/index.html]

Prettify Themes Gallery

This page requires JavaScript

# Gallery of themes for code prettify

Click on a theme name for a link to the file in revision control.
Print preview this page to see how the themes work on the printed page.
